# Assessment of Artificial Intelligence Platforms With Regard to Medical Microbiology Knowledge: An Analysis of ChatGPT and Gemini

**DOI:** 10.7759/cureus.60675

**Published:** 2024-05-20

**Authors:** Jai Ranjan, Absar Ahmad, Monalisa Subudhi, Ajay Kumar

**Affiliations:** 1 Microbiology, All India Institute of Medical Sciences, Bathinda, Bathinda, IND; 2 Animal Genetics and Breeding, Faculty of Veterinary Science and Animal Husbandry, Birsa Agricultural University, Ranchi, IND; 3 Microbiology, Institute of Medical Sciences and SUM-II Hospital, Bhubaneswar, IND; 4 Microbiology, Manipal Tata Medical College, Manipal Academy of Higher Education, Manipal, IND

**Keywords:** learning, large language model, artificial intelligence, medical education, microbiology

## Abstract

The performance of two artificial intelligence (AI) platforms, ChatGPT 3.5 (OpenAI, California, United States) and Gemini (Google AI, California, United States) was assessed by answering 200 questions of microbiology drawn from validated sources. The questions were selected from topics such as General Microbiology, Immunology, and Microbiology Applied to Infectious Diseases. The study was conducted from December 2023 to March 2024, and the responses of the different AI platforms were compared with an answer key. Statistical analysis was performed to assess accuracy. ChatGPT 3.5 and Gemini had comparable accuracy with correct response scores of 71% and 70.5%, respectively. Their performance varied across different sections. Gemini performed better in General Microbiology and Immunology, and ChatGPT 3.5 had a better score in the Applied Microbiology section. The study's findings highlight that AI platforms such as ChatGPT and Gemini can be utilized in microbiology and medical education. The evolution and continuous updating of AI platforms are required to improve their performance.

## Introduction

Microbiology or Infectious disease knowledge is vital for any graduate of medicine. This has been highlighted in recent years with outbreaks such as coronavirus disease 2019 (COVID-19), Monkeypox, Nipah, and Zika viruses. A deeper understating of such infectious diseases is of utmost importance for students, as it provides them with the tools to mitigate and manage such diseases and outbreaks in the future.

Artificial intelligence (AI) is the utilization of machines and robots to resemble human thought processes and even go beyond it. There has been significant advancement in this field since the early days of its development [1]. AI has various applications in microbiology and even medical education. Machine learning and neural networks have been employed in the recent past for the identification of microbes and cultures [2].

Medical education has also evolved in the past decade and there has been increased involvement of AI and machine learning tools. These can be utilized in formulating questions, assessing a viable solution for different cases, and providing accurate responses to different problems [3].

OpenAI (San Francisco, California, United States) is an AI research organization that has devised various platforms, such as the Generative Pretrained Transformer (GPT), which can aid in medical education and revolutionize it. This is a large language model (LLM) [4]. OpenAI launched ChatGPT (GPT-3.5) and GPT-4 [4]. These are deep learning tools that perform natural language processing. Google AI (Mountain View, California, United States) has Gemini (previously known as Bard), which is an LLM tool that can be accessed to generate responses to various questionnaires pertaining to medical education. This, in turn, will be of help in devising new strategies and tools to improve medical education technologies [5].

These platforms or chatbots can be assessed for their capability of answering difficult questions that require an in-depth knowledge of a particular subject. Even though there are a few studies evaluating the performance of ChatGPT-3.5, there are fewer studies that compare the performance of GPT-3.5 and Gemini.

In this study, we aim to assess and analyze the performance of GPT-3.5 and Gemini through a validated set of microbiology questions. This will enable us to gain a deeper insight into the functioning of these LLMs and their future use in medical education of microbiology.

## Materials and methods

This comparative study was conducted from December 2023 to March 2024. Since this study did not involve human participants, ethical clearance was not required for the study as per prevailing guidelines.

Study tool

A questionnaire comprising 200 multiple-choice questions was drawn from Ananthnarayan and Paniker’s Textbook of Microbiology [6]. The questionnaire was divided into three sections: General Microbiology (50 questions), Immunology (50 questions), and Microbiology Applied to Infectious Diseases (100 questions). A few sample questions are given in the Appendices. The creation process involved two assistant professors (JR and AK) who selected the MCQs and created the answer keys. Subsequently, a senior professor (MS) evaluated the content, ensuring accuracy, completeness, and relevance to the project objectives.

Data collection

The present study assessed free, open-source AI tools ChatGPT 3.5 and Gemini from January 2024 to March 2024. The questions were analyzed via a chat session preceded by the statement, “Please choose the best correct answer and explain your reasoning.” Each question was analyzed using a new chat session to avoid memory retention bias. To remove bias for every answer, the second response after using the regenerate response was considered for both tools. The results obtained from both the AI tools were copied and pasted into a Microsoft Word file (Microsoft Corporation, Redmond, Washington, United States) and were later evaluated using the answer key for each correct answer. One mark was assigned for each correct answer, whereas for every wrong answer zero was assigned. The scores were recorded in a Microsoft Excel sheet (Microsoft Corporation) by the first investigator (JR), and then the entire results were cross-checked by the second investigator (AK) and were finally cross-checked by the external investigators for any discrepancies in the results. The reasoning for the answers by the AI tools was not analyzed, as it would have introduced bias in results.

Statistical analysis

The data was presented in terms of frequencies and percentages, and a chi-square test was conducted online to assess the relationship between correct responses and the usage of ChatGPT 3.5 and Gemini. Statistical analysis was carried out using IBM SPSS Statistics for Windows, Version 25.0 (Released 2017; IBM Corp., Armonk, New York, United States) with significance set at P ≤ 0.05 for all tests.

## Results

On analysis, out of the 200 questions, 141 (71%) and 142 (70.5%) correct responses were given by ChatGPT 3.5 and Gemini, respectively (Table 1). Out of the 50 General Microbiology questions, ChatGPT 3.5 answered 31 (62%) correctly, while Gemini gave correct responses to 36 (72%). Thirty-four (68%) out of 50 Immunology questions were answered correctly by ChatGPT 3.5, while Gemini gave 35 (70%) correct responses in that section. ChatGPT 3.5 and Gemini provided correct answers for 77 (77%) and 70 (70%) questions, respectively, in the Applied Microbiology section.

**Table 1:** Overall comparison of correct responses from ChatGPT 3.5 and Gemini

Section	Total Questions	Correct Answers by ChatGPT 3.5, n (%)	Correct Answers by Gemini, n (%)
General Microbiology	50	31 (62%)	36 (72%)
Immunology	50	34 (68%)	35 (70%)
Microbiology Applied to Infectious Diseases	100	77 (77%)	70 (70%)
Overall Result	200	142 (71%)	141 (70.5%)

Further, we found that both platforms showed heterogeneity in understanding the questions; 58% of the questions (116 out of 200) were correctly answered by both ChatGPT 3.5 and Gemini. ChatGPT 3.5 provided the correct answers for 13% (26/200) of the questions while Gemini answered 12.5% (25 out of 200) correctly. Notably, 16.5% of the questions (33/200) remained wrong on both platforms (Chi-square with Yates correction χ2=27.65, p-value<0.00001). The overall results comparison is given in Table 2.

**Table 2:** Overall results comparison of ChatGPT 3.5 and Gemini Chi-square with Yates correction χ2=27.65, p-value<0.00001

	Answered correctly by Gemini	Answered wrong by Gemini	Row Total
Answered correctly by ChatGPT 3.5	116	26	142
Answered wrong by ChatGPT 3.5	25	33	58
Column Total	141	59	200

In the General Microbiology section, 58% (29/50) of questions were correctly answered by both ChatGPT 3.5 and Gemini (Table 3). ChatGPT 3.5 provided the correct answers for 4% (2/50) of the questions and Gemini answered 12.5% (7/50) of the questions correctly, while 24% (12/50) were answered incorrectly by both platforms (Chi-square with Yates correction χ2= 16.082, p-value=0.000061).

**Table 3:** Comparison of General Microbiology responses given by ChatGPT 3.5 and Gemini Chi-square with Yates correction χ2= 16.082, p-value=0.000061

	Answered correctly by Gemini	Answered wrong by Gemini	Row Total
Answered correctly by ChatGPT 3.5	29	2	31
Answered wrong by ChatGPT 3.5	7	12	19
Column Total	36	14	50

In the Immunology section, 56% (28/50) of questions were correctly answered by both ChatGPT 3.5 and Gemini (Table 4). ChatGPT 3.5 provided the correct answers for 12% (6/50) of the questions while Gemini answered 12.5% (7/50) questions. A total of nine out of the 50 questions (18%) were answered incorrectly by both platforms (Chi-square with Yates correction χ2=5.9918, p-value=0.014373).

**Table 4:** Comparison of Immunology responses given by ChatGPT 3.5 and Gemini Chi-square with Yates correction χ2=5.9918, p-value=0.014373

	Answered correctly by Gemini	Answered wrong by Gemini	Row Total
Answered correctly by ChatGPT 3.5	28	6	34
Answered wrong by ChatGPT 3.5	7	9	16
Column Total	35	15	50

In the Microbiology Applied to Infectious Diseases section, 59% (59/100) of questions were correctly answered by both ChatGPT 3.5 and Gemini (Table 5). ChatGPT 3.5 provided the correct answers for 18% (18/100) of the questions and Gemini alone answered 11% (11/100) of the questions correctly, while 12% (12/100) were wrong by both platforms (Chi-square with Yates correction χ2=5.6895, p-value=0.017066).

**Table 5:** Comparison of Microbiology Applied to infectious Diseases responses given by ChatGPT3.5 and Gemini Chi-square with Yates correction χ2=5.6895, p-value=0.017066

	Answered correctly by Gemini	Answered wrong by Gemini	Row Total
Answered correctly by ChatGPT 3.5	59	18	77
Answered wrong by ChatGPT 3.5	11	12	23
Column Total	70	30	100

## Discussion

The utilization of AI in medical education has risen rapidly. Various LLMs have been in use or can be used to make learning more interactive for students [7]. The implementation of self-directed learning (SDL) in Indian medical colleges by the National Medical Commission of India has paved the way for the use of such models to enhance student learning and enrich it. Students can clear their doubts and have a productive discussion with these platforms [3].

Various LLM tools have been assessed for their performance on various data sets and questions of various examinations. Gilson et al. evaluated the performance of ChatGPT with four different data sets with accuracies ranging from 42% to 64% [8]. In another study by Kung et al., an accuracy of 46% was achieved by ChatGPT 3 on a set of sample questions of the United States Medical Licensing Examination (USMLE) [9].

Microbiology is an integral part of the medical curriculum and is very crucial for understanding the etiology and pathogenesis of various infectious diseases. An assessment of ChatGPT’s knowledge of the subject was undertaken by Dipmala Das et al. [3] and Suh Huh [10], wherein the accuracy was found to be about 80% and 60%, respectively.

In our study, we analyzed the response of ChatGPT 3.5 and Gemini platforms to a standard set of microbiology questions [6]. Correct responses from ChatGPT 3.5 and Gemini platforms were found to be 71% and 70.5%, respectively. ChatGPT 3.5 had a better proportion of correct responses for applied microbiology questions than Gemini (Table 5), while for general microbiology and immunology, Gemini fared better (Tables 3, 4).

The results of our study signify that these platforms are comparable in their ability to give correct responses to the questions of Microbiology. Still, there is a difference in their accuracy for different sections of microbiology. This corroborates the findings of Das et al., where there was also a difference in the scores obtained by the AI platforms among various competency modules of microbiology [3]. This could be due to the fact that the level of difficulty among the various questions might vary, and this could affect the ability of the language models to select the correct response. Thus, these platforms need to be modified and updated regularly.

The limitation of our study is the utilization of only microbiology questions to assess the knowledge of AI platforms with a small data set. This would make it difficult to generalize to microbiology as a whole and to other domains. Additionally, the responses of these platforms were not assessed with questions of varied levels of difficulty and neither were the chatbots evaluated for their responses to subjective questions.

## Conclusions

Different AI platforms like ChatGPT and Gemini are useful in medical education. The comparable performance of these platforms to various microbiology questions shows that these can be utilized in the future for a better learning experience. With more advancements and changes in these models with time, their use in microbiology education will be beneficial.

## Appendices

Sample questions from each section

1) General Microbiology

Which microscope is based on the principle that differences in the refractive indices of bacterial cells and the surrounding medium make them clearly visible?

a) Dark-ground microscope

b) Phase-contrast microscope

c) Electron microscope

d) Confocal microscope

2) Immunology

In transplant reaction:

a) White graft response is due to delayed rejection

b) Chronic graft rejection responds well to corticosteroids

c) Acute rejection occurs within 10 days of transplant

d) All of these

3) Microbiology Applied to Infectious Diseases

A paramedical worker gets a needle prick while handling a known HIV seropositive patient. His serum was sent for testing 15 days after exposure. What is the best testing method?

a) Western blot

b) P24 capture assay

c) RT-PCR

d) Viral culture by co-cultivation
